# Finite element analysis of basicervical femoral neck fracture treated with proximal femoral bionic nail

**DOI:** 10.1186/s13018-023-04415-y

**Published:** 2023-12-06

**Authors:** Xiaodong Cheng, Yanjiang Yang, Jian Zhu, Guimiao Li, Wei Chen, Juan Wang, Qi Zhang, Yingze Zhang

**Affiliations:** 1 Department of Orthopaedics, Trauma Emergency Center, The Third Hospital of Hebei Medical University, No. 139 Ziqiang Road, Shijiazhuang, Hebei 050051 People’s Republic of China; 2 Orthopaedic Research Institute of Hebei Province, Shijiazhuang, Hebei 050051 People’s Republic of China; 3 Key Laboratory of Biomechanics of Hebei Province, Shijiazhuang, Hebei 050051 People’s Republic of China; 4 Shanxi Bethune Hospital, Shanxi Academy of Medical Science, No. 99, Longcheng Street, Taiyuan, 030032 Shanxi Province People’s Republic of China; 5 NHC Key Laboratory of Intelligent Orthopaedic Equipment, Shijiazhuang, Hebei 050051 People’s Republic of China; 6 Hebei Orthopaedic Clinical Research Center, Shijiazhuang, Hebei 050051 People’s Republic of China

**Keywords:** Finite element analysis, Basicervical femoral neck fracture, Proximal femoral bionic nail

## Abstract

**Background:**

Dynamic hip screws (DHS) and proximal femoral nail anti-rotation (PFNA) were recommended for basicervical femoral neck fracture (BFNF), however, with high rate of postoperative femoral neck shortening. The proximal femoral bionic nail (PFBN) was designed to decrease the postoperative complications associated with DHS and PFNA. The aim of this study is to compare the biomechanical characters of DHS, PFNA, and PFBN for fixation of BFNF.

**Methods:**

Using finite element analysis, we created a three-dimensional model of the BFNF for this investigation. The PFBN group, the PFNA group and the DHS + DS group were our three test groups. For each fracture group, the von Mises stress and displacements of the femur and internal fixation components were measured under 2100 N axial loads.

**Results:**

The PFBN group demonstrated the lowest stress on the implants, significantly lower than the PFNA and DHS + DS groups. In terms of stress on the implants, the PFBN group exhibited the best performance, with the lowest stress concentration at 112.0 MPa, followed by the PFNA group at 124.8 MPa and the DHS + DS group at 149.8 MPa. The PFBA group demonstrated the smallest displacement at the fracture interface, measuring 0.21 mm, coupled with a fracture interface pressure of 17.41 MPa, signifying excellent stability.

**Conclusions:**

Compared with DHS and PFNA, PFBN has advantages in stress distribution and biological stability. We believe the concept of triangle fixation will be helpful to reduce femoral neck shortening associated with DHS and PFNA and thus improve the prognosis of BFNF.

## Background

Basicervical femoral neck fracture (BFNF) is positioned between the femoral neck and the intertrochanteric region, constituting approximately 1.8% to 7.7% of all hip fractures [1–3]. BFNF exhibits a larger fracture angle and experiences higher forces and moments transmitted through the hardware in comparison with intertrochanteric fractures. Consequently, it can be regarded as a more unstable fracture compared to intertrochanteric fractures [4]. Considering that immobilization can result in severe complications, including pneumonia, urinary tract infections, pressure sores, and venous thromboembolism [5], early surgical intervention becomes imperative for the majority of these patients [6]. Nonetheless, BFNF remains labeled as an “unresolved fracture” due to its frequent association with avascular necrosis or non-union even following surgical treatment [7].

Dynamic hip screws (DHS), cannulated cancellous screws (CCS) and proximal femoral nail anti-rotation (PFNA) are commonly used devices in the treatment of BFNF [8]. However, the outcomes have been inconsistent. Mousapour et al. [9] observed that CCS fixation, typically employed for intracapsular fractures, exhibits limited effectiveness when applied to BFNF. Moreover, even the addition of extra cancellous screws to DHS to enhance its anti-rotational properties did not yield favorable outcomes. While PFNA imposes a smaller bending moment on the implant, which helps prevent further fracture site collapse and minimizes bone loss compared to DHS, its efficacy in addressing BFNF remains suboptimal. This is primarily due to the relatively narrow cortical base of the proximal fragment and the subsequent limited contact area at the primary fracture site, compounded by inadequate cancellous interdigitation [10, 11].

To address these issues, we introduced the proximal femoral bionic nail (PFBN), which draws inspiration from the well-established Gamma nail and employs a cross-structured configuration of the fixating screw and supporting screw, mirroring the proximal femur's cantilever beam structure. In light of the above, the objective of this study was to conduct a comparative analysis of the biomechanical properties of the proximal femoral bionic nail (PFBN), the dynamic hip screw with de-rotation screw (DHS + DS), and the proximal femoral nail anti-rotation (PFNA) for the treatment of BFNF. We postulated that the PFBN would exhibit superior biomechanical properties.

## Methods

This study was reviewed and approved by the institutional review board, and the informed consent was obtained from the volunteer before the examination.

### Finite element model establishment

**Fig. 1:**
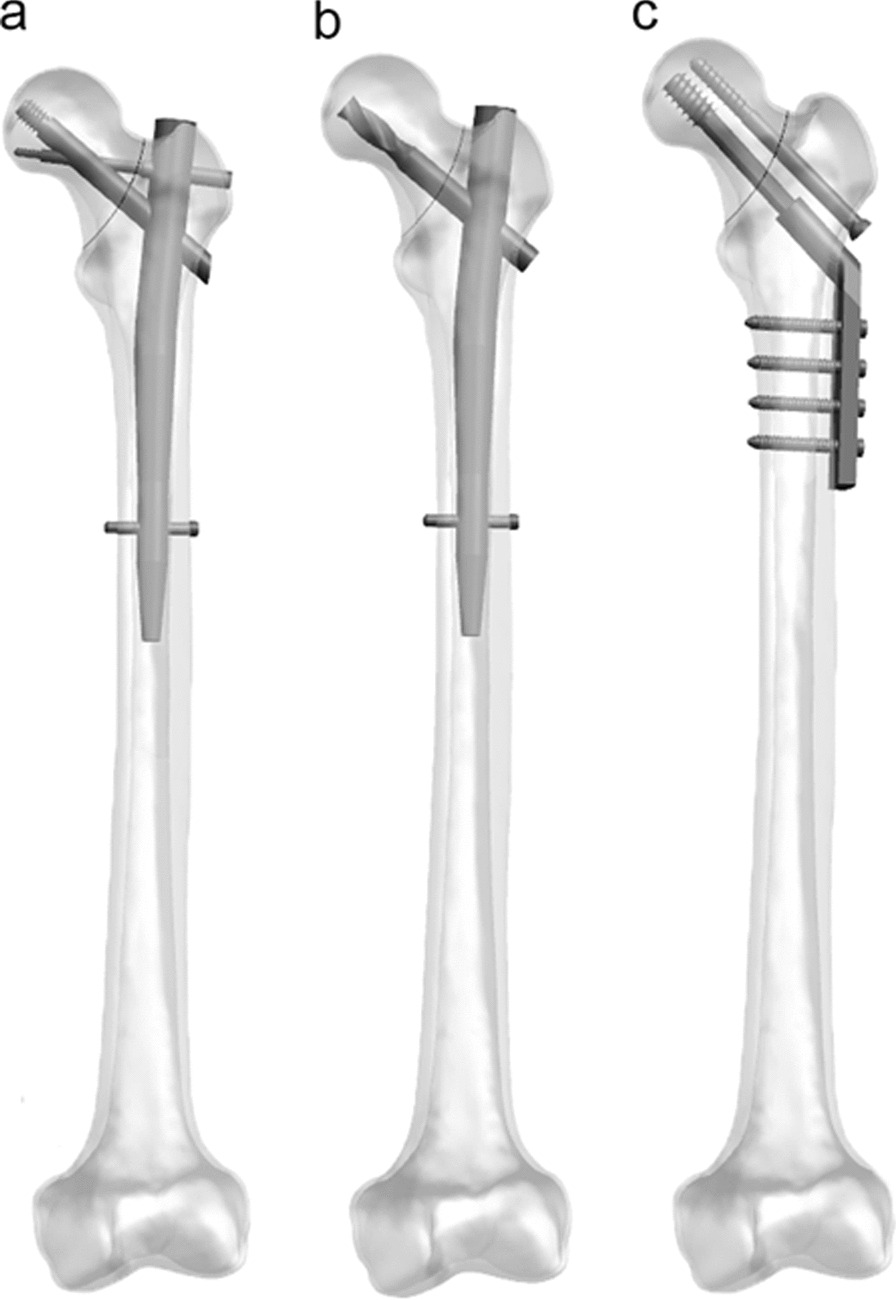
The femur model with three types of implants: **a** proximal femoral bionic nail (PFBN), **b** proximal femoral nail anti-rotation (PFNA) and **c** dynamic hip screws with de-rotation screw (DHS + DS)

**Fig. 2:**
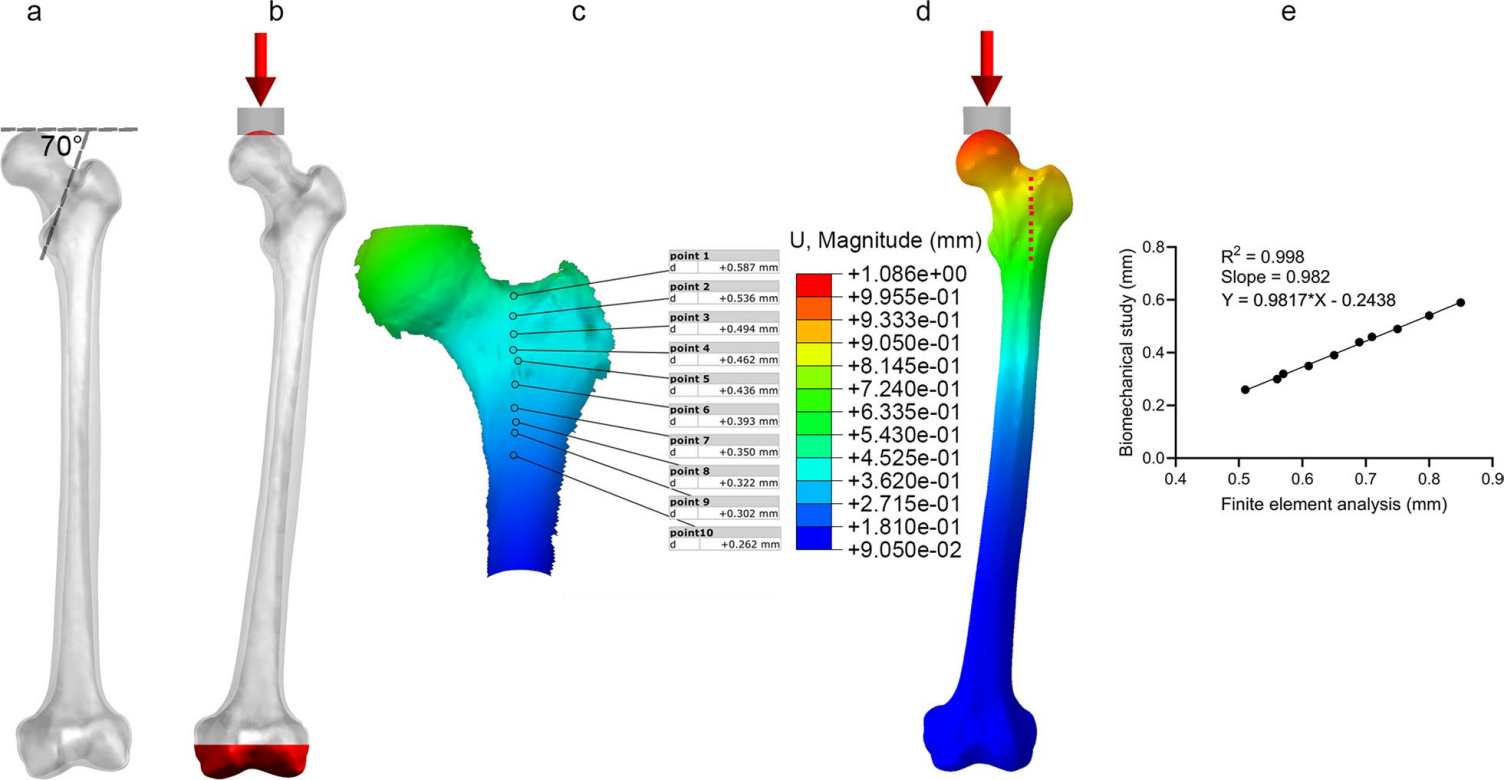
**a** Femoral neck fracture model, **b** boundary and loading conditions, **c** biomechanical verification, **d** finite element verification and **e** correlation analysis of finite element model validation

A healthy 35-year-old man with a body weight of 75 kg was recruited as a volunteer participant. After excluding the deformity and abnormal condition of hip by X-ray examination, the left femur was scanned with the spiral computed tomography scanner (Sensation 64, Siemens Medical Solutions, Forchheim, Germany). The scanning involved a slicing distance of 0.625 mm, and the raw data were stored as DICOM format. After that, the raw data were inputted into Mimics20.0 software (Materialise, Leuven, Belgium) to establish the 3-dimension model and then the non-uniform rational basis spline (NURBS) was built by using the Geomagic Studio13.0 software (Geomagic Company, USA). For accuracy, the authors employed manual segmentation to delineate the regions of cortical and cancellous bone within the CT images. The thickness of cortical bone varied across different regions due to anatomical differences. In our study, the cortical thickness ranged from 7.6 mm (femoral shaft) to 1.3 mm (femoral head). The Hypermesh 2014 software (Altair Company, USA) was used to mesh the solid model of femur with C3D4 elements. The models of the DHS + DS, PFNA and PFBN were constructed in UG-NX 12.0 (Siemens Product Life cycle Management Software Inc, USA) according to implants provided by the manufacturer (Naton Institute of Medical Technology, China). BFNF models were established and fixed with three devices by the UG-NX 12.0 (Fig. 1). According to the latest 2018 AO/OTA classification [12], the primary fracture line in the BFNF model (classified as 31-B3) is located at the base of the femoral neck, forming a 70-degree angle with the horizontal plane (Fig. 2a).

### Material properties

The material properties of femur bone model were considered to be linear elastic isotropic. In addition, titanium alloy was assigned to the implant material. The material parameters of each component are presented in Table 1 [13].

**Table 1:** Material parameters

Part	Young’s modulus, *E* (GPa)	Poisson’s ratio, *ν*
Cortical bone	17	0.3
Cancellous bone	1.15	0.25
Titanium alloy	110	0.3

### Boundary and loading conditions

A single-cycle load condition of 2100 N was applied to the finite element models using distributed coupling conditions, ensuring the uniform distribution of individual force over the bone tissue surface corresponding to the area of the femoral head [14] (Fig. 2b). The direction was normal standing angle vertical down, and the distal end of the femur was completely fixed.

The contact conditions were set as friction contact, the friction coefficient between bone and bone was 0.46, the friction coefficient between bone and nail was 0.30, and the friction coefficient between nail and nail was 0.23 [15].

### Finite element models validation

The von Mises stress on the intact femur was tested to analyze the mesh convergence. The convergence criterion used was a change of < 5%. The mesh size was set to 2 mm. To emphasize the mechanical performance of the implant in the specific region of interest, a mesh size of 1 mm was employed for all implant components. The femur was composed of 69,734 nodes and 282,872 elements (Table 2).

**Table 2:** Amounts of nodes and elements of four components

Components	Nodes	Elements
Normal femur	69,734	282,872
PFBN	132,114	545,788
PFNA	123,227	513,870
DHS + DS	113,281	459,533

The ElectroForce 3330 Series II (TA Instruments, USA) was employed to apply axial pressure ranging from 0 to 600 N onto the surface of the femoral head at a rate of 5 N/s. Concurrently, the high-speed camera integrated within the GOM non-contact optical strain measurement system (GOM GmbH, Germany) captured the loading process at a frame rate of 7 frames/s. The resultant images were subsequently subjected to computer processing to derive displacement images and quantify displacement specific to the femur under an axial pressure of 600 N. Subsequent to data acquisition, the GOM Software 2021 was employed to select the appropriate starting point for calculations based on the collected images and to define the calculation area. Upon completion of the calculations, the displacement cloud diagram was automatically generated (Fig. 2c).

Under the same loading and boundary conditions as the biomechanical experiment, the displacement at the corresponding position were calculated for the normal femur finite element model (Fig. 2d). The correlation analysis result indicates that our model is appropriate for the subsequent study (Fig. 2e).

## Results

### Stress on the implanted femur

**Fig. 3:**
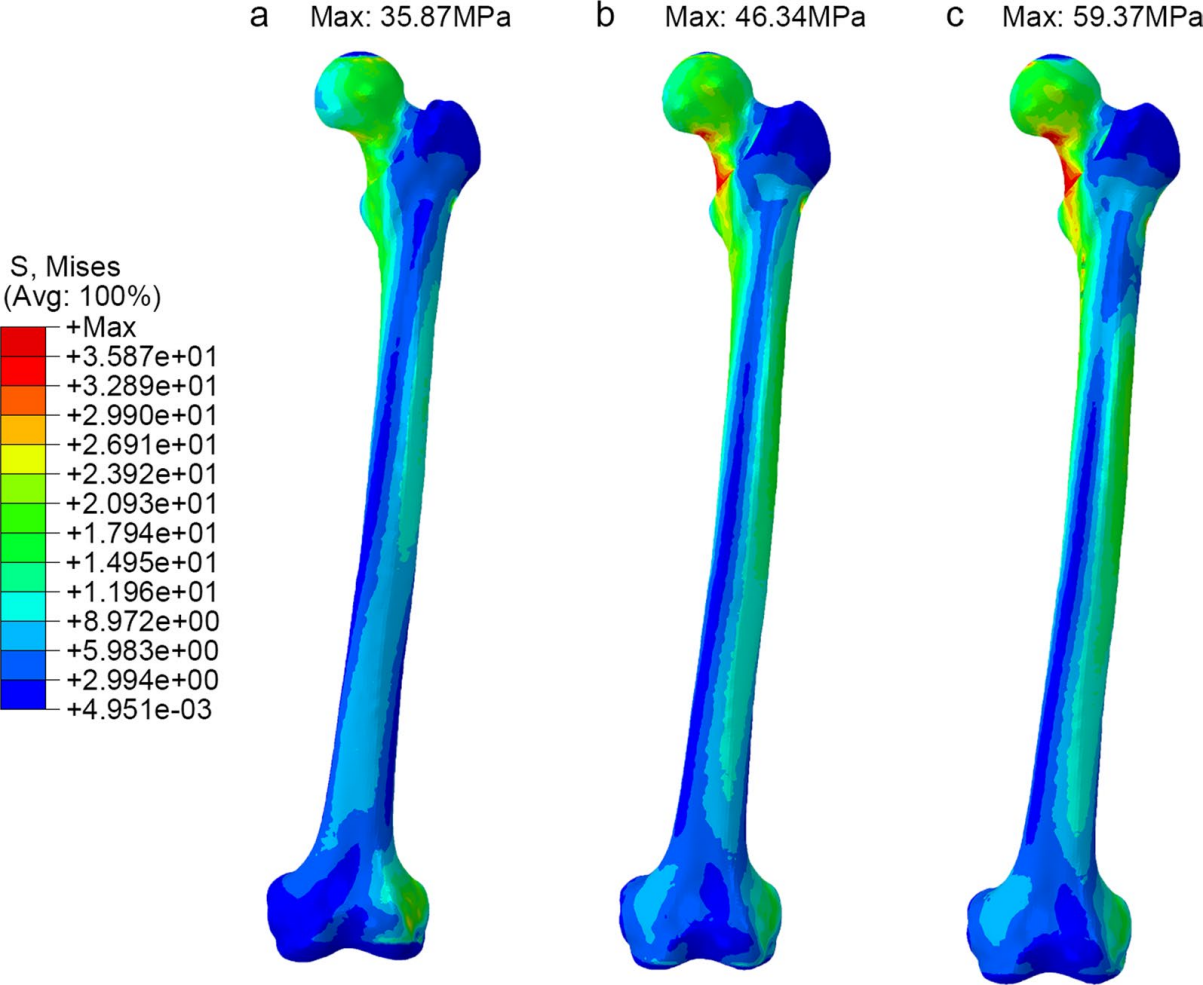
The von Mises stress distribution of femur in three models (**a** PFBN model, **b** PFNA model, **c** DHS + DS model)

Figure 3 compares the peak values of the maximum von Mises stress in the three fixation models. Among the three models, the PFBN group exhibited the lowest overall femoral stress, measuring 35.87 MPa. The maximum von Mises stress was observed beneath the inner cortical bone of the femoral neck. Similarly, in the PFNA group, which utilizes intramedullary fixation, the maximum stress was concentrated beneath the inner cortical bone of the femoral neck, measuring 46.34 MPa. The DHS + DS group experienced the highest stress, reaching 59.37 MPa, with the maximum von Mises stress appearing at the distal end screw hole in the outer cortical bone.

### Stress on the implants

**Fig. 4:**
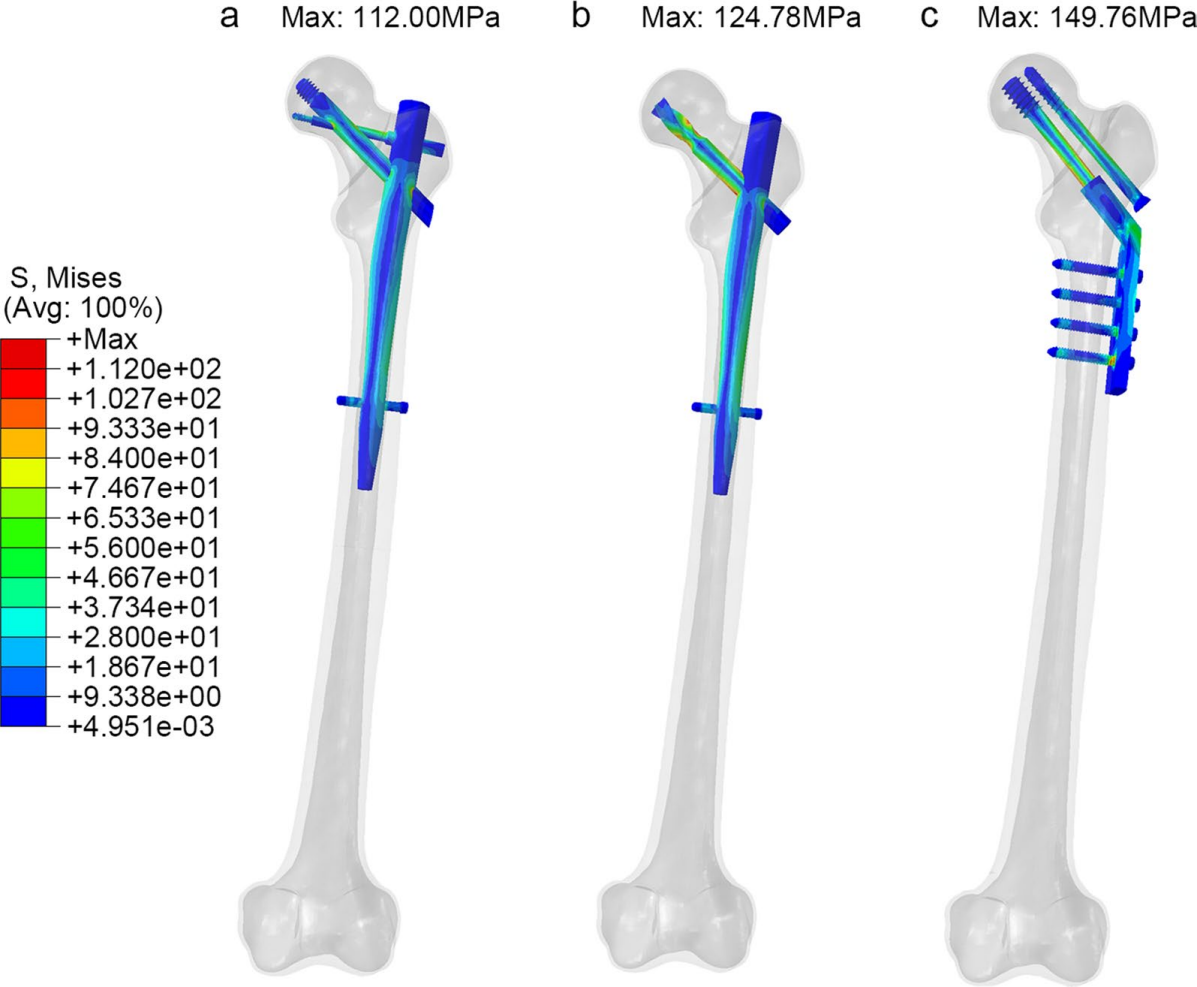
The von Mises stress distribution of implant (**a** PFBN model, **b** PFNA model, **c** DHS + DS model)

To assess the performance of the implant components, Fig. 4 compares the maximum von Mises (equivalent) stress in three fixation styles. The DHS + DS group exhibited the highest internal fixation stress at 149.8 MPa, with the stress concentrated primarily on the shaft of the lag screw. In the PFNA group, the maximum internal fixation stress reached 124.8 MPa, primarily concentrated around the screw-blade interface. The PFBN group experienced a maximum internal fixation stress of 112.0 MPa, with the highest stress concentration occurring at the intersection of the fixating screw and the main nail, significantly lower than the internal fixation stress in the DHS + DS and PFNA groups.

### Model displacement of the femur

**Fig. 5:**
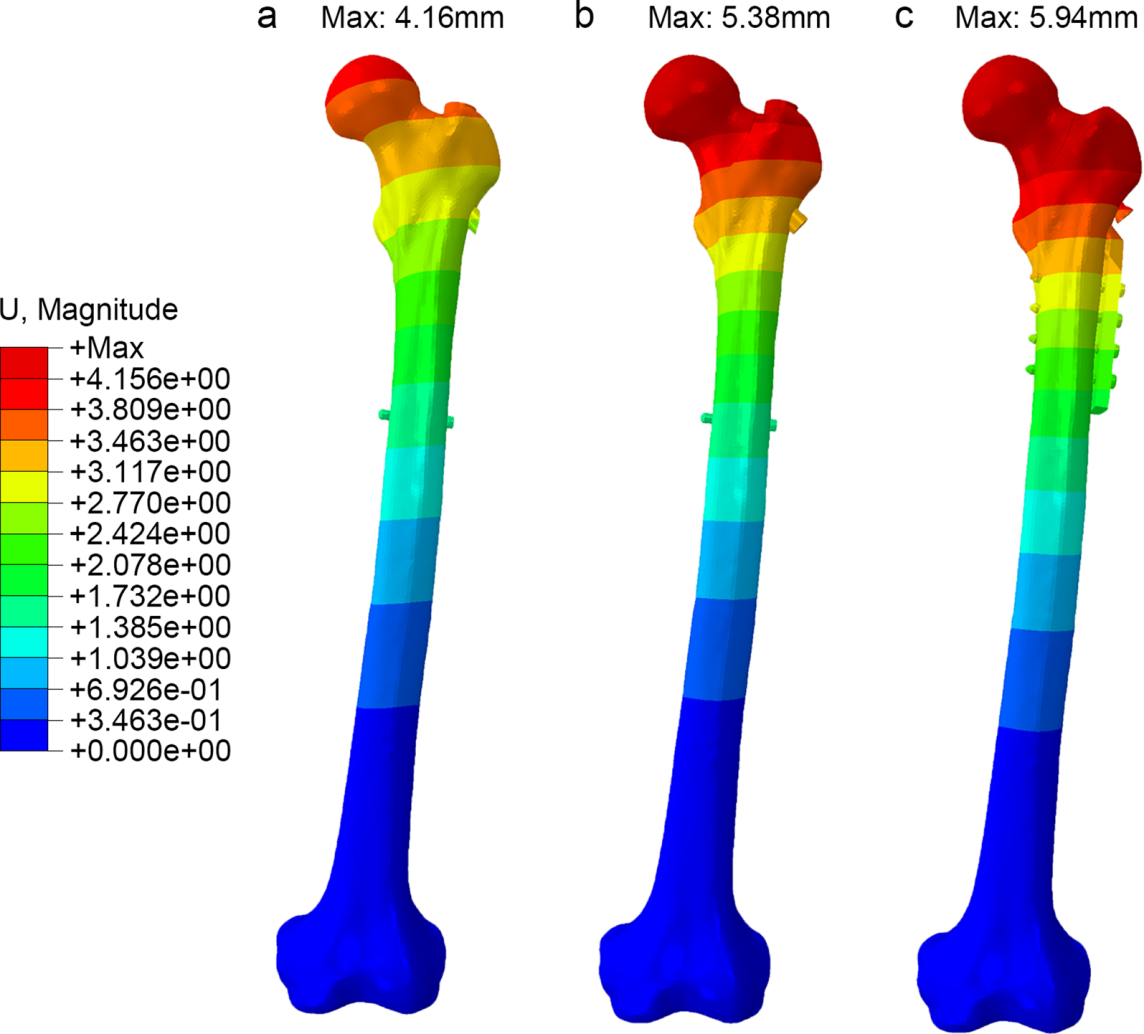
The displacement distribution of three models (**a** PFBN model, **b** PFNA model, **c** DHS + DS model)

The deformation of the femoral models in the three groups (Fig. 5) primarily occurred in the proximal femoral region. The DHS + DS group exhibited an overall femoral model displacement of 5.939 mm, while the PFNA group had an overall displacement of 5.377 mm. In the PFBN group, the overall femoral model displacement measured 4.156 mm.

### Model displacement and contact pressure of fracture surfaces

**Fig. 6:**
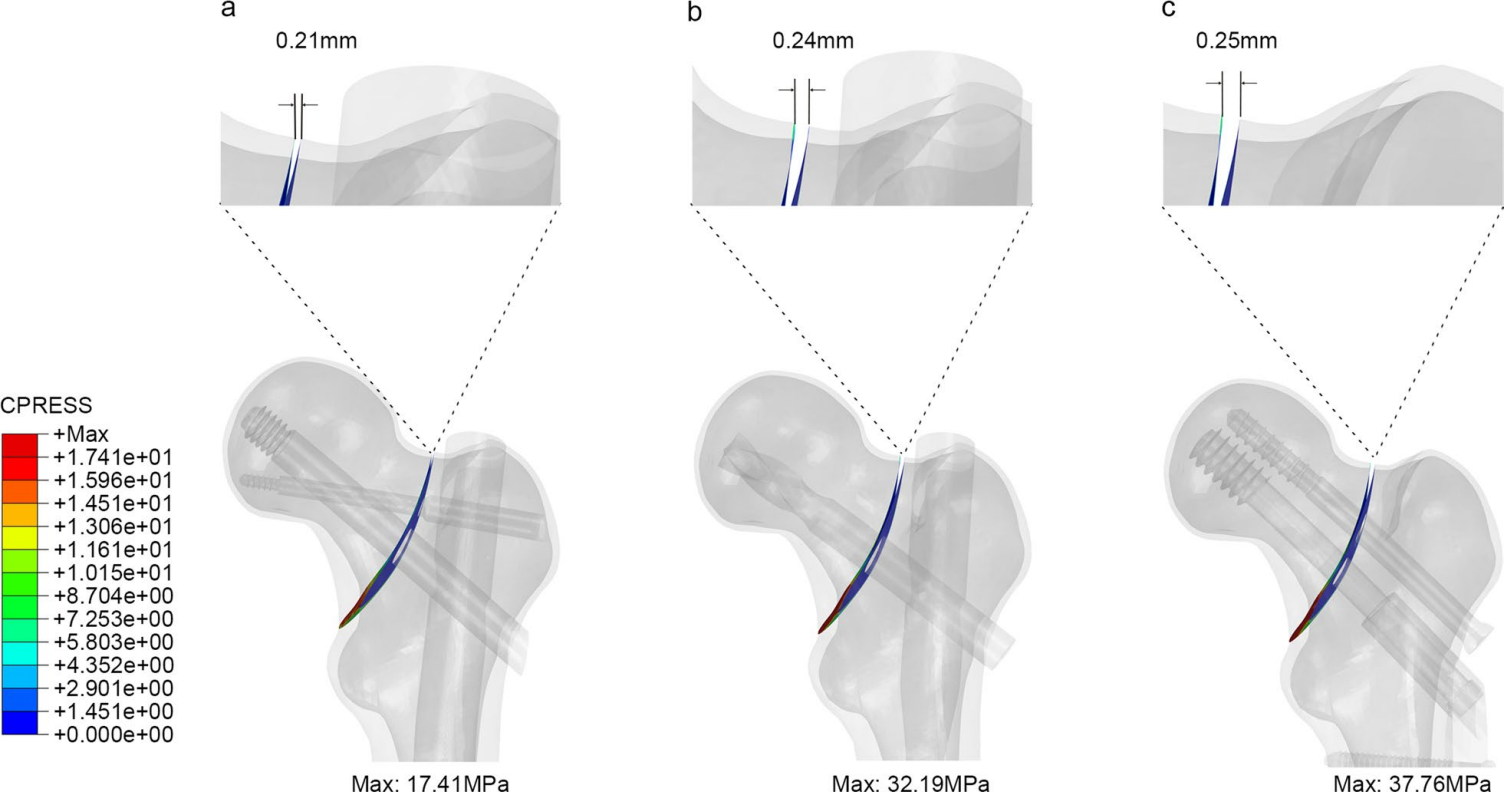
The displacement and contact pressure at the fracture interfaces of the three models (**a** PFBN model, **b** PFNA model, **c** DHS + DS model)

Figure 6 illustrates the displacement and contact pressure at the fracture interfaces of the three models. At the fracture interface, the PFBN group exhibited the smallest displacement of 0.212 mm and a fracture interface pressure of 17.41 MPa, demonstrating excellent stability. In the PFNA group, the fracture face displacement measured 0.241 mm, with a fracture interface pressure of 32.19 MPa. The DHS + DS group displayed relatively lower stability, with a fracture interface displacement of 0.254 mm and a fracture interface pressure of 37.76 MPa.

## Discussion

In the present study, we investigated the mechanical properties of PFBN, DHS + DS and PFNA for the treatment of vertical BFNF using finite element analysis. Our study found that the primary stability of BFNF fixed with PFBN was significantly improved compared with DHS + DS and PFNA. Besides, the peak stress and stress distribution of PFBN and proximal femur were lower than that of DHS + DS and PFNA. PFBN demonstrated better ability to resist shearing force of BFNF, which may be crucial to improve the clinical outcomes of BFNF.

Currently, the selection of the most suitable implant for internal fixation in the treatment of BFNF is a subject of ongoing debate. Osteosynthesis using DHS or CCS is the standard care [16–18]. However, the postoperative femoral neck shortening has raised growing concerns. This was due to weakness of abductor muscles and inferior hip function resulted from the subsequent decrease of abductor lever arm. In addition, femoral neck shortening after BFNF can increase the risk of femoral head collapse [19]. Given that BFNF treated by sliding implants is not as stable as previous believed, various devices or techniques for length-stability of femoral neck have been developed [20–22]. The use of intramedullary fixation system has been suggested by some authors [23, 24]. In a retrospective clinical study, Guo [25] reported that intramedullary nails had a trend to decrease the femoral neck shortening compared with CCS in treatment of unstable BFNF (5.0% vs 14.29%). Other researchers have investigated the biomechanical properties between cephalomedullary nails and DHS. In a comparative study with synthetic femora, Imren et al. [26] found the PFNA has higher failure loads and possesses biomechanical benefits for fixation of unstable basicervical fractures compared with DHS. Nevertheless, Seyhan and colleagues discovered a heightened likelihood of encountering the following conditions within the PFNA group: reverse displacement of the proximal screw, proximal femur shortening, and a reduction in the varus angle of the proximal femur [27]. When managing unstable hip fractures in geriatric patients, PFNA demonstrates a mechanical failure rate of 7.5% [28]. This encompasses a range of complications, including implant cut-out with an incidence between 5.4 and 13% [29, 30], coxa vara occurring at a rate of 2.5% [31], and a 1% incidence of internal fixation failure [32]. Due to the substantial risk of a reverse wedge effect associated with PFNA fixation for basicervical fractures, this may not effectively mitigate neck collapse or prevent mechanical cut-out failure, even with the enhanced rotational stability provided by the helical blade [33].

The occurrence of various mechanical failures may be attributed to the mismatch between these implants and the proximal femoral anatomical structure and mechanical transmission. In their investigation of complications associated with internal fixation, Zhang et al. have suggested that a triangular stabilization structure (Chinese patents: ZL200920254063.4, ZL200920254062.x, ZL201120370391.8) could potentially reduce the likelihood of internal fixation failure, thereby contributing to the development of proximal femoral bionic nail (PFBN). The innovation of PFBN lies in its double triangle structure, composed of the supporting screw, fixating screw, and main nail. This design closely replicates the cantilever beam structure typically found in a normal proximal femur, resulting in a significant improvement in the postoperative stability following BFNF. The first component, known as the mixed triangle, is formed by the combination of the supporting screw, fixating screw, and the cancellous bone of the femoral head. This configuration significantly enhances the rotational stability of the hardware within three-dimensional space, ensuring a stable transfer of body-weight load to the junction of the supporting screw and fixating screw. The second component, referred to as the metal triangle, is constructed from the fixating screw, supporting screw, and main nail. The combined triangle and the main nail together create a stable cantilever beam structure that aligns with the anatomical structure and mechanical characteristics of the femoral neck. Moreover, the fixating screw is supported by both the main nail and the supporting screw, resulting in a double-pivot fixation. Consequently, this shortens the force arm, ultimately reducing stress concentration and enhancing fracture stability. Moreover, the tension stress of fixating screw can be significantly shared by the supporting screw due to horizontal placement. In our study, the PFBN group reduced stress on the implanted femur by 39.6% and 22.6% compared to the DHS and PFNA groups, respectively.

As shown in displacement distribution, the fracture section stability and overall construct stability were higher in the PFBN model than that of DHS + DS and PFNA models, which meant that PFBN had good ability to resist compression and tension force. The peak stress of DHS + DS and PFNA was 1.3 and 1.1 times higher than that of PFBN. We believe the supporting screw plays an important role in reducing the stress concentration of fixation screw in PFBN, which could decrease the incidence of screw withdrawal, cut-out, and hip varus. In elderly patients with BFNF, who often have osteoporosis, PFBN could potentially offer improved stability and support for early postoperative rehabilitation exercises. However, it should be noted that our study did not specifically investigate the mechanical performance of PFBN in an osteoporotic model, and further research is needed to validate these assumptions.

In addition, the peak stress of proximal femur in the PFBN model had the least value among all models, which demonstrated that PFBN had a decreased dependence on the integrity of femoral medial cortex. However, the peak stress and stress concentration of proximal femur in DHS + DS and PFNA models were located at the medial cortex of femur, which was different from that of PFBN. We consider that the results could be partially explained by the poor construct stability of the DHS + DS and PFNA. In a study on fracture morphology of BFNF patients, Collinge et al. [34] found 96% of cases had femoral neck comminution, which was located at the inferior in most cases (94%). Therefore, the PFBN is a suitable internal fixation for treating the BFNF, especially associated with comminution of medial cortex. Taken together, our study indicated that PFBN can not only enhance the mechanical stability of BFNF model, but also make improvement in the stress distribution of implant and proximal femur.

### Limitation

To our knowledge, our study is the first study to test the mechanical properties of PFBN in BFNF. However, there are some limitations to this study. Firstly, despite superior biomechanical stability of PFBN, we did not verify whether the use of PFBN would result in better clinical outcomes. Further randomized comparative studies are needed to verify the clinical benefit. Additionally, it is important to consider that patients with osteoporosis may exhibit lower mechanical properties than healthy patients, potentially leading to higher displacements in such cases.

## Conclusions

In conclusion, we investigated the mechanical properties of PFBN, DHS and PFNA in the treatment of BFNF by using finite element analysis. Our study indicated that PFBN had better ability to resist shearing force and could improve the mechanical character compared with DHS and PFNA. Therefore, the use of PFBN should be considered in BFNF. Our findings provide a basis for further research and clinical utility of PFBN in BFNF patients.

## Data Availability

Please contact author for data requests.

## Abbreviations

FEA: Finite element analysis
BFNF: Basicervical femoral neck fracture
DHS: Dynamic hip screws
DS: De-rotation screw
PFBN: Proximal femoral bionic nail
PFNA: Proximal femoral nail anti-rotation
